# RNA-seq Analysis of Early Hepatic Response to Handling and Confinement Stress in Rainbow Trout

**DOI:** 10.1371/journal.pone.0088492

**Published:** 2014-02-18

**Authors:** Sixin Liu, Guangtu Gao, Yniv Palti, Beth M. Cleveland, Gregory M. Weber, Caird E. Rexroad

**Affiliations:** USDA/ARS National Center for Cool and Cold Water Aquaculture, Kearneysville, West Virginia, United States of America; INRA, France

## Abstract

Fish under intensive rearing conditions experience various stressors which have negative impacts on survival, growth, reproduction and fillet quality. Identifying and characterizing the molecular mechanisms underlying stress responses will facilitate the development of strategies that aim to improve animal welfare and aquaculture production efficiency. In this study, we used RNA-seq to identify transcripts which are differentially expressed in the rainbow trout liver in response to handling and confinement stress. These stressors were selected due to their relevance in aquaculture production. Total RNA was extracted from the livers of individual fish in five tanks having eight fish each, including three tanks of fish subjected to a 3 hour handling and confinement stress and two control tanks. Equal amount of total RNA of six individual fish was pooled by tank to create five RNA-seq libraries which were sequenced in one lane of Illumina HiSeq 2000. Three sequencing runs were conducted to obtain a total of 491,570,566 reads which were mapped onto the previously generated stress reference transcriptome to identify 316 differentially expressed transcripts (DETs). Twenty one DETs were selected for qPCR to validate the RNA-seq approach. The fold changes in gene expression identified by RNA-seq and qPCR were highly correlated (R^2^ = 0.88). Several gene ontology terms including transcription factor activity and biological process such as glucose metabolic process were enriched among these DETs. Pathways involved in response to handling and confinement stress were implicated by mapping the DETs to reference pathways in the KEGG database.

**Accession Numbers:**

Raw RNA-seq reads have been submitted to the NCBI Short Read Archive under accession number SRP022881.

**Customized Perl Scripts:**

All customized scripts described in this paper are available from Dr. Guangtu Gao or the corresponding author.

## Introduction

Aquaculture is the fastest-growing animal food producing sector of agriculture. Rainbow trout (*Oncorhynchus mykiss*) is not only an important aquaculture species; it is also a primary research model for fish [1]. Fish under intensive rearing conditions experience various stressful conditions such as handling, crowding, sub-optimal water quality and temperature fluctuations. Stress has been shown to have negative impacts on survival, growth, reproduction and fillet quality [2], [3], [4], [5], [6], [7], [8], [9], therefore to improve production efficiency it is crucial to understand stress responses at the physiological and molecular levels. To this end stress responses have been extensively studied in fishes [4], [10], [11], [12].

The stress response is initiated by the activation of the hypothalamus-pituitary-interrenal (HPI) axis and release of catecholamines from chromaffin cells that induce many biochemical and physiological changes [4]. The advent of microarray technologies made it possible to observe global changes in gene expression in response to stress and these stress hormones [12], [13], [14]. For instance, a custom microarray was used to study gene expression changes after handling [15], and the 16K microarray for salmonid species was used to study the transcriptome response to heat stress in the red blood cells of rainbow trout [16]. Although the use of microarrays was a significant advance in exploring the transcriptome response to stress in rainbow trout [12], they have several limitations. For example, detection is limited to the probes included in the microarray design, the detection range is limited by background signal and signal saturation, and differences in expression between paralogous genes is complicated by cross-hybridization, which is a particular problem for species such as the rainbow trout where more than half of the genes are thought to be duplicated due to a relatively recent whole genome duplication [17], [18].

High-throughput next-generation DNA sequencing technologies have revolutionized analyses of genomes and transcriptomes. Transcriptome sequencing, or RNA-seq, overcomes many inherent limitations of microarray technologies, and has emerged recently as a powerful tool to characterize gene expression profiles in both model and non-model species [19], [20]. Application of RNA-seq has been used in rainbow trout by Morera et al. [21] to study the transcriptome of nucleated erythrocytes, Salem et al. [22] to identify DNA markers associated with growth, and Palstra et al. [23] to identify differentially expressed transcripts in response to exercise. Although not in rainbow trout, RNA-seq has been used to investigate stress in fish, specifically heat stress in rainbowfish (*Melanotaenia duboulayi*) [24] and catfish (*Ictalurus* spp.) [25]. Our long term goal is to identify and characterize genes involved in general and specific stress responses in rainbow trout leading to management or breeding practices that improve production efficiency. In the current study we sought to employ an RNA-seq approach to identify genes and pathways whose expression is altered due to handling and confinement stress. As a whole genome reference sequence has not yet been obtained for this species, we previously generated a stress reference transcriptome by 454 sequencing transcripts from a pool of tissues from fish exposed to a variety of stressors [26]. In the current study, we mapped Illumina sequencing reads from liver transcripts obtained from fish exposed to handling and confinement stress onto the reference transcriptome to identify differentially expressed transcripts (DETs). Also, gene enrichment and pathways responding to handling and confinement stress were identified through gene ontology and KEGG annotation.

## Materials and Methods

### Experimental animals and stress challenge

The experimental fish and the stress challenge have been reported in our previous publication [26], the current study focused solely on the handling/crowding stressor. During this challenge the fish were transferred from a 193 L holding tank to a 15 L tank, therefore we now prefer to use the term of handling and confinement to describe the combination of stressors more accurately. The experiment was conducted under approval of the USDA/ARS National Center for Cool and Cold Water Aquaculture Institutional Animal Care and Use Committee, protocol #50. Briefly, four unrelated families from the NCCCWA even year broodstock population under selective breeding for growth [27] were tagged and two fish (average boy weight ∼57 grams) from each family were placed in each of five 193 L tanks on flow-through water (eight fish per tank). After two-week acclimation, two random tanks of fish were used as experimental control and the other three tanks of fish were subjected to handling and confinement challenge using the procedures previously described by Weber and colleagues [28] as adapted from Pottinger *et al.* [29]. The fish were netted and transferred from the 193 L tank to a 15 L tank and left undisturbed for three hours followed by euthanization with a lethal dose of MS-222. Tissues (blood; brain; gill; heart; kidney; liver; spleen and muscle) were collected immediately and flash-frozen in liquid nitrogen.

### RNA extraction, library preparation and sequencing

Using the standard TRIzol protocol, total RNA was extracted from the liver of each individual fish in five tanks, including three challenged tanks and two control tanks. Equal amount of total RNA from six fish was pooled by tank, and the pooled RNA was used to prepare mRNA libraries for sequencing following the standard Illumina protocol. The five RNA-seq libraries were sequenced with a read length of 75 nucleotides in one lane of Illumina HiSeq 2000. To increase the read coverage and examine the technical sequencing variation, a total of three sequencing runs were performed.

### Identification of DETs in response to handling and confinement stress

Software CD-HIT [30] was used to remove redundant sequences among the rainbow trout stress reference transcriptome generated in our previous study [26]. There were 125,486 non-redundant reference sequences including three sets of sequences: Newbler isogroups, MIRA contigs and singletons. Five nucleotides were trimmed from each end of the Illumina reads. The trimmed reads were aligned to the non-redundant reference transcriptome using the software Bowtie [31] with a maximum of two mismatches. The output of Bowtie was filtered with an in-house Perl script to generate the count table. Only reads mapped uniquely to a reference sequence or a Newbler isogroup were included in the count table. We used the statistics package DESeq [32] incorporated in software package GENE-Counter [33] to process the count table to identify DETs. The count table was directly imported into the system and the “fit-only” option was selected for the dispersion estimation step of DESeq. To control the false discovery rate of multiple-hypothesis testing, the p-values calculated by DESeq were further processed by the R package qvalue [34] using its default setting. The final list of DETs was generated with a cutoff q-value less than 0.05.

### GO enrichment and pathway analysis

The GOSSIP [35] program implemented in the Blast2GO [36] package was used for GO enrichment analysis. The GO terms for each transcript were extracted from the GO annotation generated in our previous study [26]. The DET sequences were selected as the test set and the non-redundant reference sequences were used as the reference set. The enriched GO terms for biological process and molecular function were identified using a two-tailed Fisher's Exact Test that corrects for multiple testing with a false discovery rate less than 0.05. The web-server of KAAS [37] on the KEGG website was used to identify the k number for each transcript in the DETs list. The k numbers of DETs were used for reconstruction of the pathways and BRITE functional hierarchies to identify broad systemic functions.

### Validation of gene expression fold changes by qPCR

Twenty-one transcripts covering the full range of gene expression fold changes were selected for qPCR. The PCR primers designed with the software primer 3 are listed in Table S2. A pool of RNA samples from five fish was used to optimize the qPCR reactions for each primer pair. Equal amount of liver RNA was pooled from one random fish per tank. The pooled RNA was treated with DNase I, and the first strand of cDNA was generated with the MMLV reverse transcriptase 1^st^-strand cDNA synthesis kit following the manufacture's protocol (Progema, Madison, WI). The cDNA was diluted and used for qPCR in a total reaction volume of 15 ul containing 7.5 ul SYBR green supermix, 4 ul cDNA template and an optimized amount of primers. The optimized reactions amplify a single product with a single peak for the melting curve and a single band on agarose gel. The PCRs were also optimized with an efficiency value between 1.85 and 2.15. After qPCR was optimized, the qPCR was performed in duplicate for each individual liver RNA samples used for RNA-seq. The housekeeping gene β-actin has been widely used as a control for qPCR in rainbow trout [15], [16], [38], [39]. Also, the expression of this gene based on RNA-seq data was not different between the challenged tanks and control tanks in this study. Therefore, gene β-actin was used to normalize the expression level of each transcript. The normalized expression was calculated using the relative expression ratio method described by Pfaffl [40].

## Results

### Sequencing and mapping

In total, 491,570,566 reads were generated and have been archived in the NCBI's short read archive, accession number SRP022881 (Table 1). About 70.9% of these reads were mapped uniquely to the reference transcriptome (Table 1). The majority of reads (68.5%) were mapped onto the Newbler contigs, and 1.9% and 0.5% of reads were mapped to MIRA contigs and singletons, respectively. As the mapping results were in great agreement among runs (Fig. S1), we used the combined mapping results to identify DETs in response to handling and confinement stress.

**Table 1:** Summary of sequencing and mapping results.

Run	Total Reads	Uniquely Mapped Reads							
		Newbler Contigs		MIRA Contigs		Singletons		Total Mapped Reads	
1	196,490,154	136,242,238	69.3%	3,804,064	1.9%	927,177	0.5%	140,973,479	71.7%
2	201,869,621	137,890,026	68.3%	3,892,544	1.9%	941,603	0.5%	142,724,173	70.7%
3	93,210,791	62,760,963	67.3%	1,800,483	1.9%	422,610	0.5%	64,984,056	69.7%
**Total**	**491,570,566**	**336,893,227**	**68.5%**	**9,497,091**	**1.9%**	**2,291,390**	**0.5%**	**348,681,708**	**70.9%**

### Identification of DETs in response to handling and confinement stress

A total of 316 DETs (Table S1) were identified in response to handling and confinement stress. Only 74 DETs were down-regulated with a range of fold change from −1.6 to −12.9; the remaining 242 DETs were up-regulated with a range of fold change from 1.6 to 22.4. Among these 316 DETs, 126 DETs could not be annotated with functional descriptions due to lack of sequence similarities with well-characterized genes in the NCBI database. The top 20 up-regulated and down-regulated transcripts for which functional annotation was assigned are listed in Table 2. It is notable that multiple DETs have the same sequence description where the expression levels of closely related genes have been delineated, such as multiple hits for genes which are similar to insulin-like growth factor binding protein 1.

**Table 2:** Top 20 up-regulated and down-regulated transcripts with functional annotation.

Accession	Length	Fold change	Sequence description
g17813.1	1415	−11.88	MHC class II alpha chain
MIRA_rep_c1368.1	558	−6.33	heat shock protein 30
g03437.1	4357	−5.25	heat shock protein 70
g07619.1	2231	−4.80	neurofilament light polypeptide
g18799.1	1306	−3.97	proto-oncogene c-fos
g14497.1	2731	−3.83	arrestin domain containing protein 3
g00004.40	1066	−3.62	enolase 2
g14619.1	2495	−3.29	multidrug and toxin extrusion protein 1
GMRRXZN02HWWQU.1	474	−3.28	kit receptor
g25669.1	829	−3.05	reproduction regulator 2
g00783.1	2541	−3.05	tublin subunit alpha
g23019.1	981	−3.00	C-type lectin domain family 4, member E
g24283.1	904	−2.48	VIP peptides precursor
g18360.1	1357	−2.48	protein phosphatase 1
g01667.1	826	−2.38	complement c1q-like protein 4 precursor
g20378.1	1166	−2.32	acidic mammalian chitinase precursor
g04310.2	687	−2.31	toxin-1 precursor
GKYT6YL01BJ6WJ.1	455	−2.31	vitellogenin c
g04310.1	1091	−2.29	toxin-1 precursor
MIRA_c24695.1	468	−2.24	matrilin 3
g23318.1	962	18.47	papilin-like protein
MIRA_c15889.1	492	14.70	mitochondrial phosphate carrier member 25
MIRA_rep_c838.1	383	14.67	cell surface protein
g07186.1	483	14.00	UPF0686 protein c11orf1-like
g05769.1	1418	13.98	diablo mitochondrial precursor
g19034.1	1278	12.34	transmembrane protein 80
g17253.1	1492	7.93	chitinase 1
g22613.1	1002	7.89	calcium-binding mitochondrial carrier protein
g15569.1	1881	7.33	pepsinogen c
g00268.2	1420	7.10	zymogen granule membrane protein 16 homolog -like
GKYT6YL02FT55P.1	405	6.91	transcription factor cp2-like protein 1
g00268.1	1509	6.75	zymogen granule membrane protein 16 homolog -like
g06628.1	1210	6.41	coenzyme q-binding protein coq10 homolog
g16976.1	1540	6.40	regulator of G-protein signaling 1
g10133.2	708	6.02	insulin-like growth factor binding protein 1
g17943.1	1399	5.77	insulin-like growth factor binding protein 1
g10133.1	1313	5.68	insulin-like growth factor binding protein 1
g39048.1	463	5.53	transcription factor cp2-like 1
g05419.1	489	5.33	barrier-to-autointegration factor
g00766.1	2319	5.22	tsc22 domain member 3

### GO (gene ontology) enrichment and pathway analysis

GO terms were assigned to 158 out of the 190 DETs that were annotated by sequence similarity to well characterized genes in the NCBI database. For GO enrichment analysis, we focused on biological process and molecular function; 36 enriched GO terms were identified. Most of these were represented by a few DETs. GO terms represented by 10 or more DETs are shown in Table 3. In terms of molecular function, only transcription factor activity (GO:0003700 and GO:0001071) was significantly enriched. There were six enriched GO terms for biological process (Table 3) including response to stimulus (GO:0050896), response to organic substance (GO:0010033), negative regulation of programmed cell death (GO:0043069), negative regulation of cell death (GO:0060548), regulation of cell growth (GO:0001558) and glucose metabolic process (GO:0006006).

**Table 3:** Enriched GO terms in response to handling and confinement stress.

GO ID	GO term	Category^1^	FDR	P-value
GO:0003700	sequence-specific DNA binding transcription factor activity	F	0.0256	1.62E-05
GO:0001071	nucleic acid binding transcription factor activity	F	0.0256	1.65E-05
GO:0050896	response to stimulus	P	0.0369	5.80E-05
GO:0010033	response to organic substance	P	0.0496	1.21E-04
GO:0043069	negative regulation of programmed cell death	P	0.0369	5.72E-05
GO:0060548	negative regulation of cell death	P	0.0435	7.85E-05
GO:0001558	regulation of cell growth	P	0.0435	7.20E-05
GO:0006006	glucose metabolic process	P	0.0496	1.23E-04

1 F: molecular function; P: biological process.

Using the web-based server KAAS, we were able to assign 78 k numbers to 91 DETs (Table S1). Based on the result of BRITE reconstruction, the two largest groups were enzymes with 33 DETs and transcription factors with 14 DETs. We also mapped these 91 DETs with k numbers to the reference pathways in KEGG database. Even though these 91 DETs were mapped to many reference pathways, none of the pathways had more than 7 matched k numbers. Nonetheless, several pathways such as MAPK signaling pathway, glycolysis/gluconeogenesis and insulin signaling pathway, were implicated in our analysis. The DETs involved in those pathways were discussed in detail below (see discussion).

### Validation of DETs using qPCR

**Figure 1:**
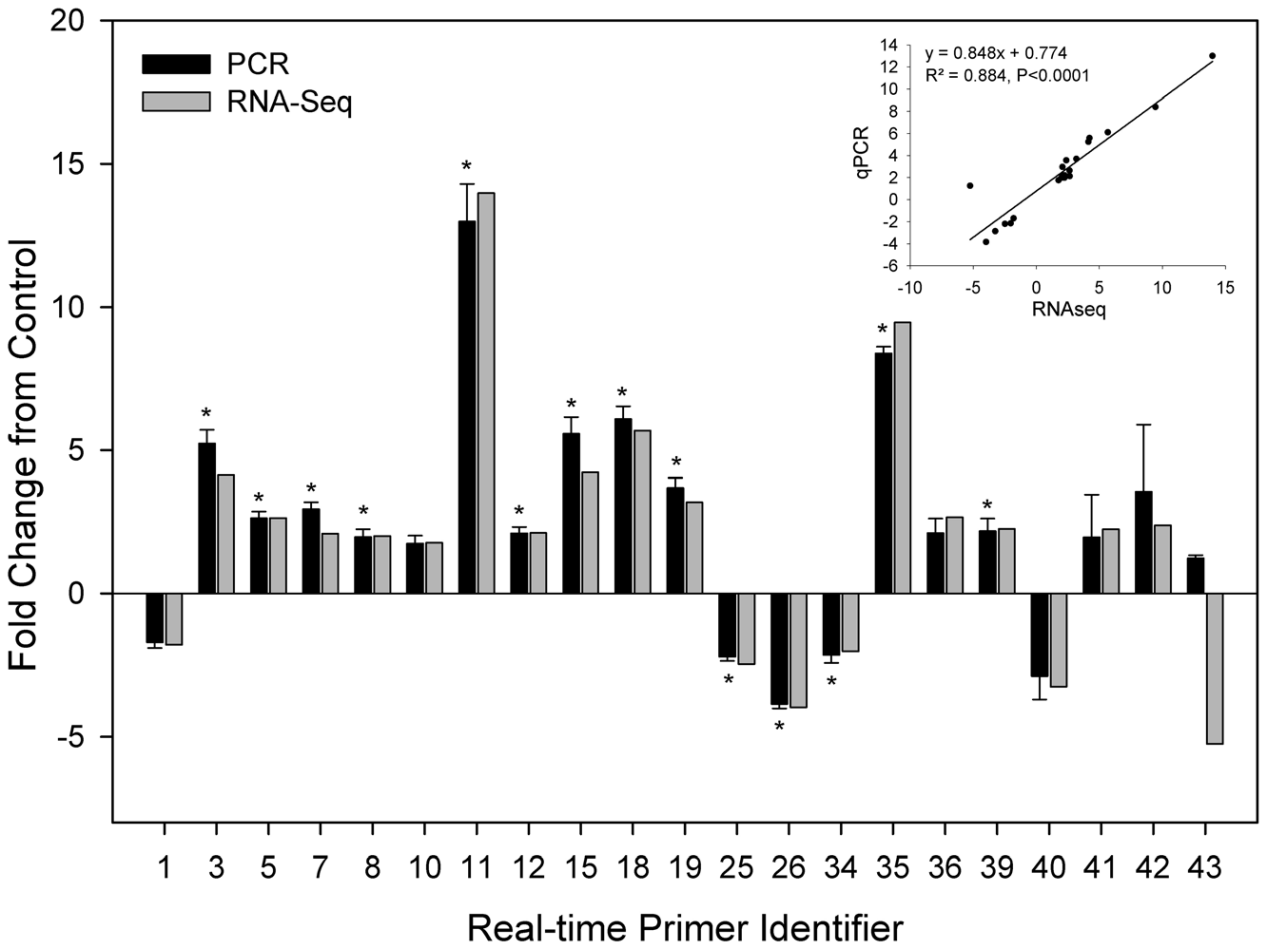
Comparison of transcript expression in terms of fold change as measured by RNA-seq and qPCR. The transcript expression fold changes measured by RNA-seq and qPCR are indicated by dark grey and light grey columns, respectively. Asterisks on the qPCR values indicate significant differences between control and stressed fish at *p*<0.05.

Twenty-one DETs covering the full range of expression fold changes were selected for qPCR to validate the results of RNA-seq analysis. For all 21 DETs except one (primer pair RT43), both the direction and magnitude of gene expression changes were similar between those measured by qPCR and RNA-seq analysis (Fig. 1). The transcript expression fold changes measured by these two methods were highly correlated with a significant coefficient of determination of 0.88 (*p*-value <0.0001). However, qPCR only identified significant differences in expression for 14 transcripts (supplementary Table S2).

## Discussion

As reported in Sanchez et al. [26], fish challenged with handling and confinement stress differed significantly from controls in terms of their plasma lactate and cortisol concentrations; but no significant differences were observed with respect to their plasma glucose, chloride, or lysozyme concentrations [26]. Glucose levels in both controls and treated fish were at levels we typically see in our stressed fish with this same challenge with this same population of fish [28]. Since cortisol levels in the control group were typical of unstressed fish, and cortisol levels in the treatment group were typical of fish exposed to the same handling and confinement stress, it is unlikely glucose was elevated in the control group in response to exposure to an unintended stressor. The stress challenge elicited multiple specific physiological responses; therefore these fish were used for further analyses with RNA-seq.

### RNA-seq is a powerful tool to study stress response in rainbow trout

RNA-seq has revolutionized transcriptome studies in both model and non-model organisms, with an emphasis in the latter for providing an efficient approach for gene discovery, expression analyses, and identification of sequence polymorphisms. We report the use of RNA-seq to identify 316 DETs that respond to handling and confinement stress in rainbow trout. The reliability and accuracy of RNA-seq analysis was demonstrated by the 0.88 coefficient of determination between RNA-seq and qPCR.

Both known and novel stress response genes in fish were among the 316 DETs identified in this study. For example, Momoda et al. [41] reported that both glucose 6-phosphatase (G6Pase) and JunB were consistently up-regulated in liver of rainbow trout in response to confinement stress. These two genes were also up-regulated in the present study. Diablo (or SMAC) is a protein released from mitochondria following apoptotic stimuli and inhibits the actions of inhibitors of apoptosis proteins. Diablo expression has been shown to increase following exposure of European flounder to pollutants [42]. This gene was also highly up-regulated (13.98 fold) and its increased expression was also confirmed by qPCR in the present study. Moreover, some well documented mammalian stress response genes with little information in fish were also among the DETs. For instance, gadd45 proteins encoded by three genes, gadd45a, gadd45b and gadd45g, have been identified as stress sensors in mammals as they modulate signaling for physiological and environmental stress [43]. So far, little is known for their role in stress responses in fishes. All three genes were among the 316 DETs reported in this study. Interestingly, gadd45a was down-regulated and gadd45b and gadd45g were up-regulated. Furthermore, 126 out of the 316 DETs reported in this study could not be annotated with functional description due to lack of sequence similarity to orthologs in the NCBI database. However, some of these novel transcripts likely have critical roles in response to handling and confinement stress as their fold changes in expression ranged from −12 to 22 (Table S1).

Among the 21 transcripts selected for qPCR, twenty transcripts had very similar fold changes in expression based on RNA-seq and qPCR, respectively. Only one transcript (primer pair RT43) had an opposite expression fold change based on RNA-seq and qPCR. This transcript was annotated as hsp70, whose expression in liver has been observed to increase in response to elevated plasma cortisol concentrations that are associated with some stress responses but not including handling and confinement in rainbow trout [15], [26], [44], [45]. Although our qPCR results show a slight increase in expression (Figure 1), it is not statistically significant. Thus, we speculate that handling and confinement stress did not cause differential expression of hsp70 in our study.

Also, the qPCR results were only determined to be statistically significant for 14 transcripts (Table S2). This may demonstrate the sensitivity or high false positive rate of RNA-seq; however it also highlights the confidence gained when: 1) interpretations based on single gene analysis are validated with multiple protocols; and 2) results implicating pathways are interpreted based on differential expression of more than one gene. Validating pathways containing physiologically relevant differential expression should include identification of potential DETs in the data set not represented in the reference transcriptome; and further evaluation of genes expected to be represented in the data set and in pathways of DETs.

### Pathways implicated in response to handling and confinement stress in rainbow trout

Activation of HPI axis and catecholamine release leads to many changes at the biochemical and physiological level. Since liver is the major organ for metabolic adjustments, it is not surprising to observe enrichment of GO terms related to glucose metabolism and transcription factors in this study. Due to the limited annotation of DETs identified in this study and the paucity of well documented pathways in rainbow trout, it is challenging to describe all of the exact pathways involved in response to handling and confinement stress in rainbow trout. However, several pathways were implicated in response to handling and confinement stress in this study. Glucose is an important fuel that is oxidized to meet the energy demand associated with stress response. Elevation of plasma glucose concentration is a well-documented metabolic stress response in rainbow trout. Activation of the gluconeogenesis pathway in response to stress has been reported in several previous studies [41], [46]. Our data support this observations as GO terms for glucose metabolic processes were enriched among the 316 DETs identified in this study. In relation to the activation of gluconeogenesis pathway, we observed the up-regulation of PEPCK (phosphoenolpyruvate carboxykinase) and G6Pase. PEPCK, a rate limiting enzyme for the gluconeogenesis pathway, converts oxaloacetate into phosphoenolpyruvate and carbon dioxide. G6Pase is an enzyme catalyzing the hydrolysis of glucose-6-phosphate into glucose and plays a critical role in glucose homeostasis. Consistent with a previous report in rainbow trout that amino acids are the predominant substrate for gluconeogenesis [11], we also observed up-regulation of genes involved in protein metabolism such as cathepsin D. This is also in agreement with the reported up-regulation of cathepsin D transcript in the culture of rainbow trout hepatocytes treated with cortisol [46].

While cortisol is a key hormone associated with the stress response, other hormones such as insulin-like growth factor (IGF), insulin, and estrogen signaling may also play important roles. Similar to the increased expression of hepatic expression of IGF binding protein-1b (IGFBP-1b), -2a, and -3 during transport in zebrafish [47], we observed up-regulation of IGFBP-1 in response to handling and confinement stress. Approximately 1% of plasma IGF-I is free, while the remaining is bound to IGFBPs [48], and hepatic expression of IGFBPs largely regulates the stability and amount of free IGF-I in plasma [49]. Therefore, increased IGFBP expression likely reduces IGF-I signaling in peripheral tissues, including skeletal muscle in which IGF-I promotes protein retention [50], [51]. Thus, reduced IGF signaling in stressed fish likely leads to increased protein degradation and the subsequent mobilization of amino acids as substrates for increased gluconeogenesis. Additionally, up-regulation of IRS (insulin receptor substrate), PI3K (phosphatidylinositol 3-kinase) and down-regulation of PP1 (protein phosphatase 1), which implicates the activation of the insulin signaling pathway in response to handling and confinement stress. Consistent with the role of insulin in stress response, Wiseman et al. [15] observed transient elevation of the transcripts for insulin receptors after a brief handling disturbance of rainbow trout. In support of the role of estrogen in response to stress, we observed altered transcript abundance for vitellogenins, the major yolk proteins in most oviparous organisms. Vitellogenins are encoded by a small gene family, and their expression is under estrogenic control. Consistent with the inhibition of vitellogenin gene expression by cortisol treatment [52], we observed decreased expression of vitellogenin c in the present study. Interestingly, we also noticed elevated expression of two other vitellogenins. Taken together, these results implicate a complex interplay of cortisol and other endocrine signals in stress responses.

It is clear that the immune response was altered in rainbow trout in response to handling and confinement stress. Many important elements of innate and adaptive immunity such as cytokine receptors, antigen presenting and processing molecules, immunoglobins and transcriptional factors were among the DETs identified in this study. We observed that cytokine receptors such as c-x-c chemokine receptor type 4 (CXCR4), c-c chemokine receptor 9 (CCR9) and tumor necrosis factor receptor member 9 (SF9), were up-regulated. However, cytokine receptor KIT (proto-oncogene tyrosine protein kinase) was down-regulated. Transcription factor NF-κB might play critical roles in the regulation of diverse cellular responses including cell proliferation, survival and inflammation [53]. The activation of NF-κB is an immediate-early step of immune and anti-apoptosis process. When NF-κB is in the active state, it moves from the cytoplasm to the nucleus where it stimulates the transcription of various proinflammatory and antiapoptotic factors. NF-κB is inactivated by IκB binding, and the inactivated NF-κB is transported back to cytoplasm. We observed that IκB, inhibitor of NF-κB, was up-regulated in this study. Consistent with this observation, up-regulation of IκB in response to stress or pathogens has been reported in several studies. Lewis et al. [16] reported that IκB was significantly up-regulated in the blood of rainbow trout during recovery of heat stress. Transcription factor JunB is a member of the activator protein 1 (AP-1) transcription complex [54]. AP-1 regulates many cellular processes including cell proliferation, differentiation, apoptosis and stress response. We observed up-regulation of JunB in the liver response to handling and confinement stress, which is consistent with other studies of confinement stress response in rainbow trout [41]. Similarly, up-regulation of JunB in response to heat stress was observed in the red blood cells [16] and atrophying muscle [55] of rainbow trout. Transcription factor c-Fos is also a component of AP-1. Contrary to the traditional paradigm where JunB and c-Fos are coregulated, c-Fos was found to be down-regulated in this study; changes in expression of genes JunB and c-Fos were both confirmed by qPCR. However, annotation for these genes is largely derived from mammalian species, and the number and diversity of Jun and Fos genes in fishes suggests they may be under divergent transcriptional controls [56].

Even though induction of acute phase response in fish is often associated with the activation of the inflammatory response, acute phase response proteins (APPs) also play a role in response to confinement stress challenge in rainbow trout. Cairns et al. [38] reported the up-regulation of APP in the liver of rainbow trout after six hours of confinement stress challenge. Using qPCR, Talbot et al. [39] confirmed up-regulation of three APP genes, serum amyloid A, haptoglobin and differentially regulated trout protein 1, in the liver of rainbow trout after eight or more hours of confinement. In this study, the fish were subjected to only three hours confinement challenge, and we did not observe any of those acute phase genes among the 316 DETs identified in the present study. We speculate that this discrepancy may be caused by the shorter confinement challenge in this study. Of course, this discrepancy could also be caused by other factors such as genetic differences between brood stocks used, age of fish and other differences in environmental factors.

## Conclusions

Deep sequencing of the rainbow trout liver transcriptome in response to handling and confinement stress identified 316 DETs. The reliability and accuracy of RNA-seq analysis was demonstrated by the 0.88 coefficient of determination between RNA-seq and qPCR. Several GO terms including transcription factor activity and -biological process such as glucose metabolic process were enriched among the 316 DETs. Pathways involved in response to handling and confinement stress were implicated by mapping the DETs to the reference pathways in KEGG database. The findings of this study provide a basis for further characterization of molecular response to handling and confinement stress in rainbow trout.

## Supporting Information

Figure S1 **Comparison of normalized uniquely mapped reads among three runs. 1A, tank1; 1B, tank 2; 1C, tank 3, 1D, tank 4; and 1E, tank 5.**
(PPTX)

Table S1 **All 316 DETs in response to handling and confinement stress identified in this study.**
(XLSX)

Table S2 **Primers used for qPCR to validate transcript expression fold changes measured by RNA-seq.**
(XLSX)
