# Supplementary figures and images for: RNA-seq Analysis of Early Hepatic Response to Handling and Confinement Stress in Rainbow Trout
Source: PLoS One. 2014 Feb 18;9(2):e88492. doi: 10.1371/journal.pone.0088492 (PMC3928254; doi:10.1371/journal.pone.0088492)

## Slide 1
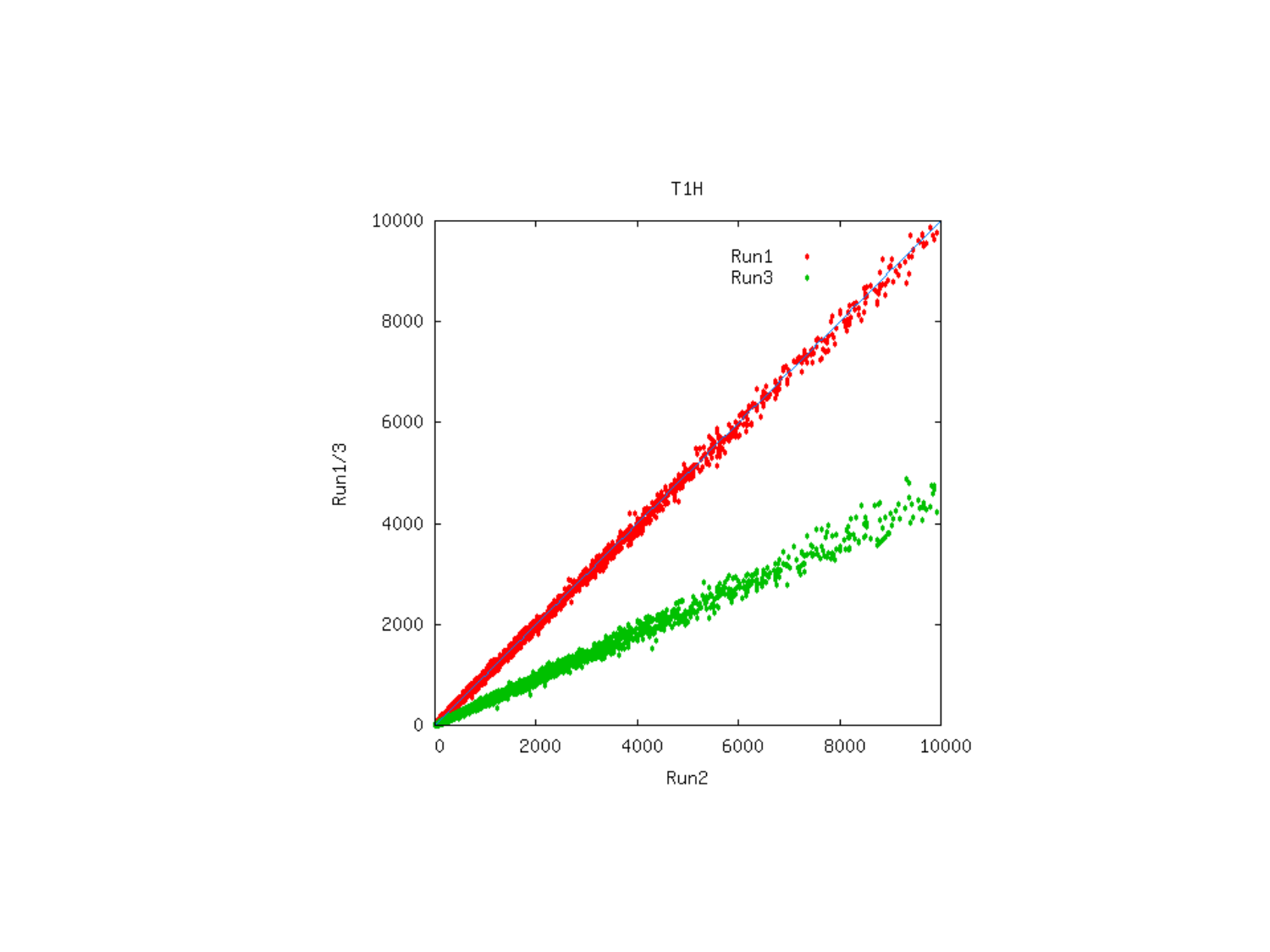

## Slide 2
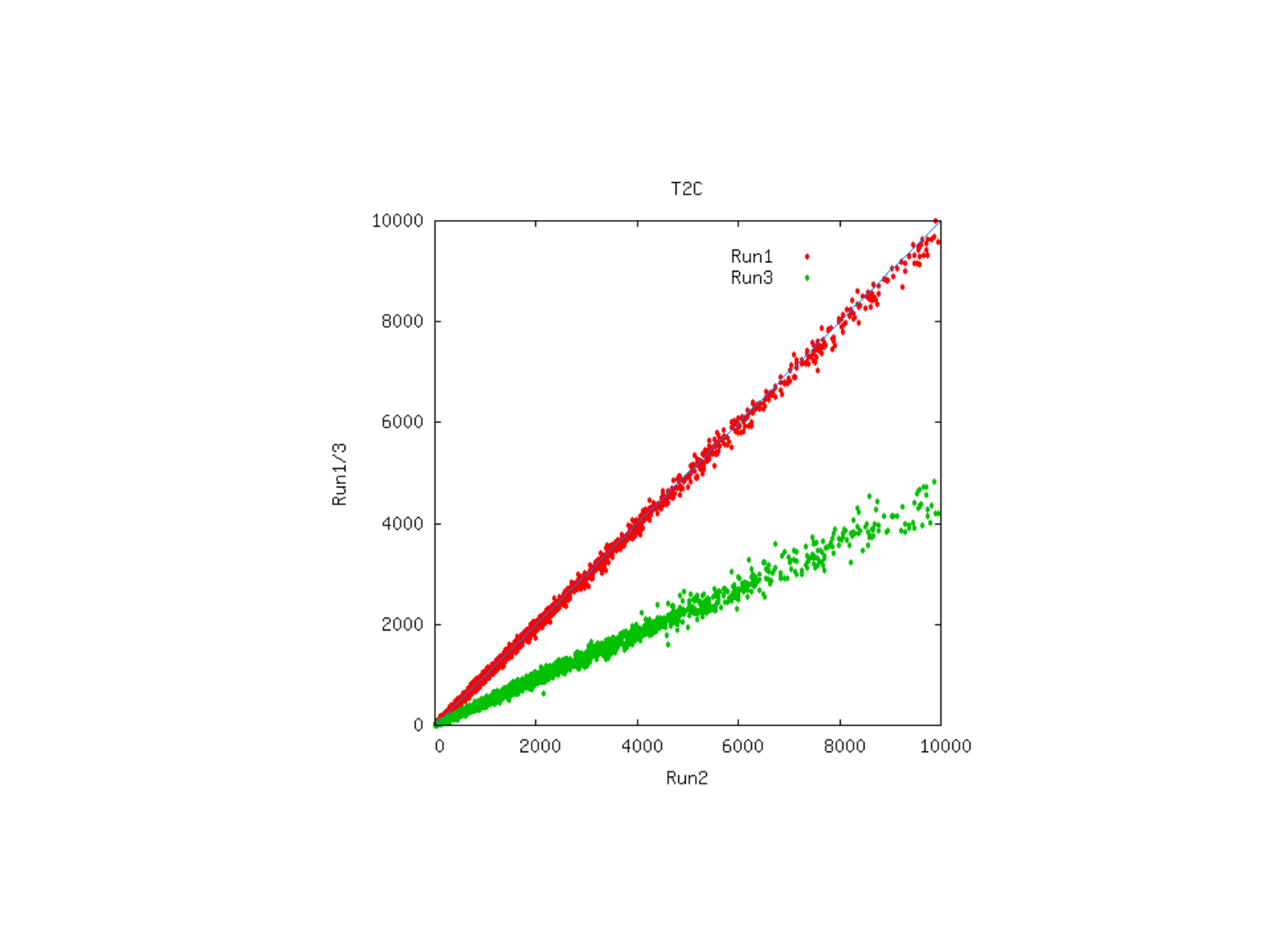

## Slide 3
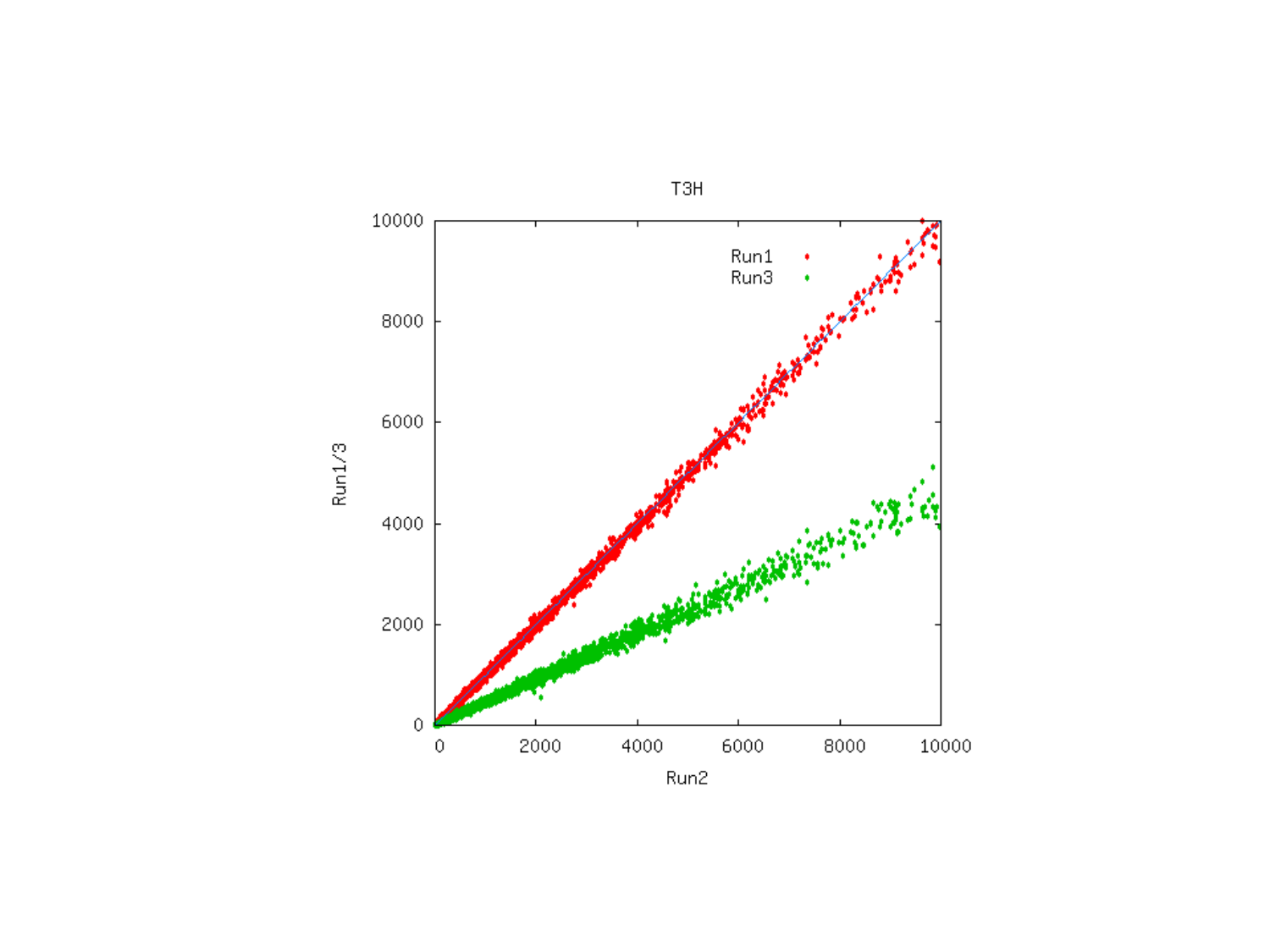

## Slide 4
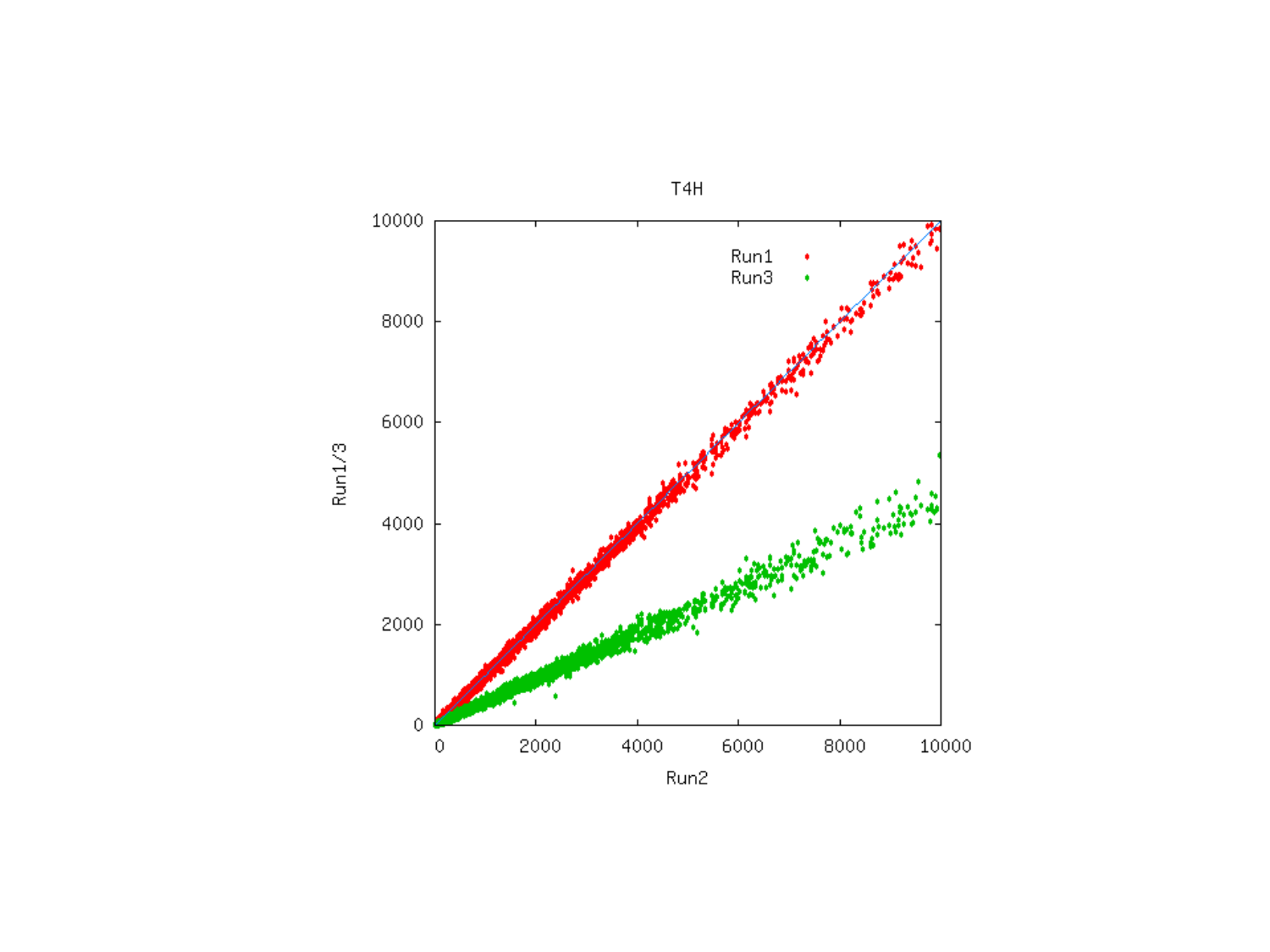

## Slide 5
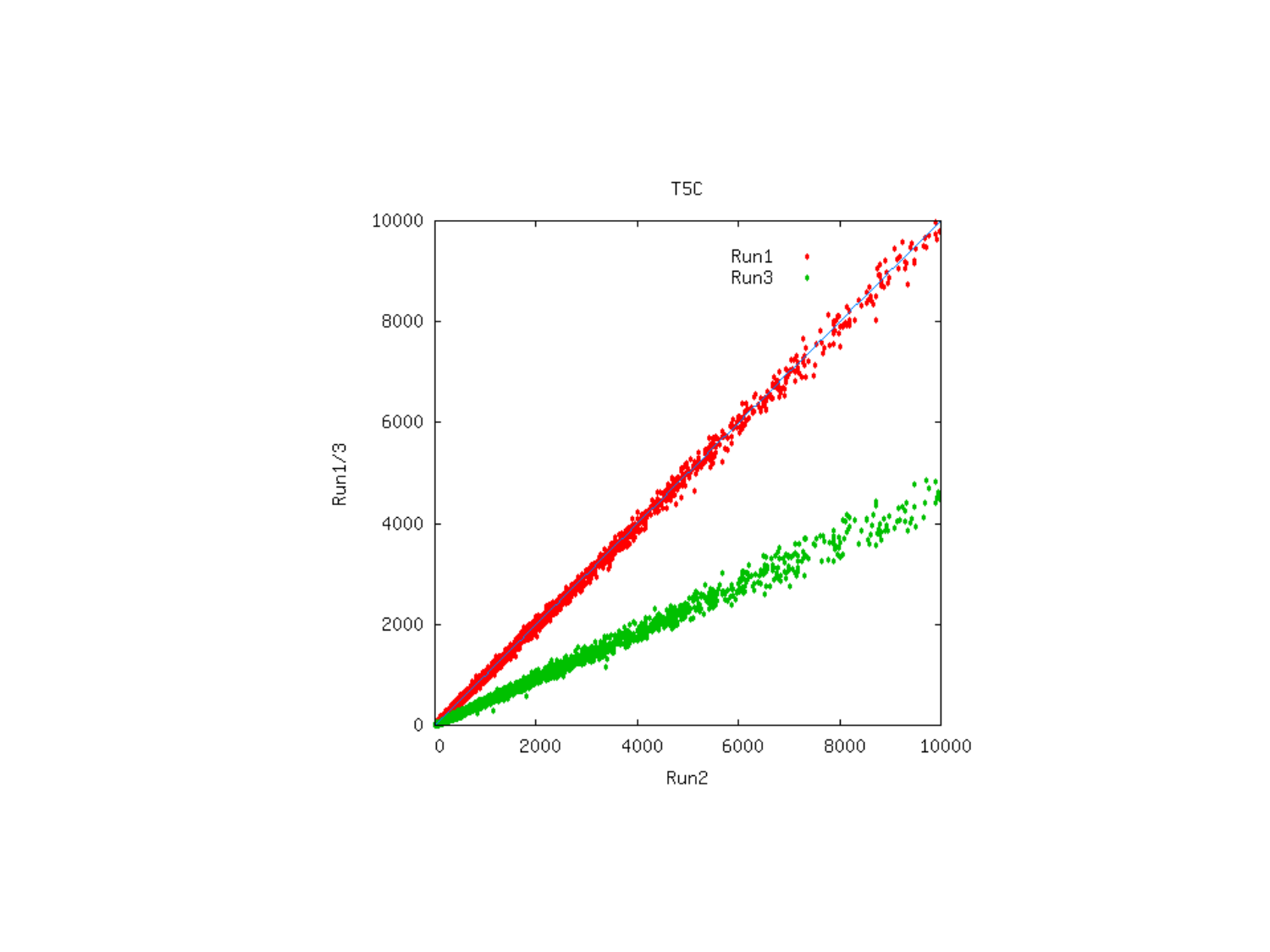

Supplement: Figure S1 — Comparison of normalized uniquely mapped reads among three runs. 1A, tank1; 1B, tank 2; 1C, tank 3, 1D, tank 4; and 1E, tank 5. (PPTX) [file pone.0088492.s001.pptx]
